# Supplementary material for: In situ X-ray diffraction and the evolution of polarization during the growth of ferroelectric superlattices
Source: Nat Commun. 2015 Dec 4;6:10136. doi: 10.1038/ncomms10136 (PMC4686826; doi:10.1038/ncomms10136)
Supplement: Supplementary Information — Supplementary Figures 1-5 and Supplementary Notes 1-5 [file ncomms10136-s1.pdf]

SUPPLEMENTARY FIGURES

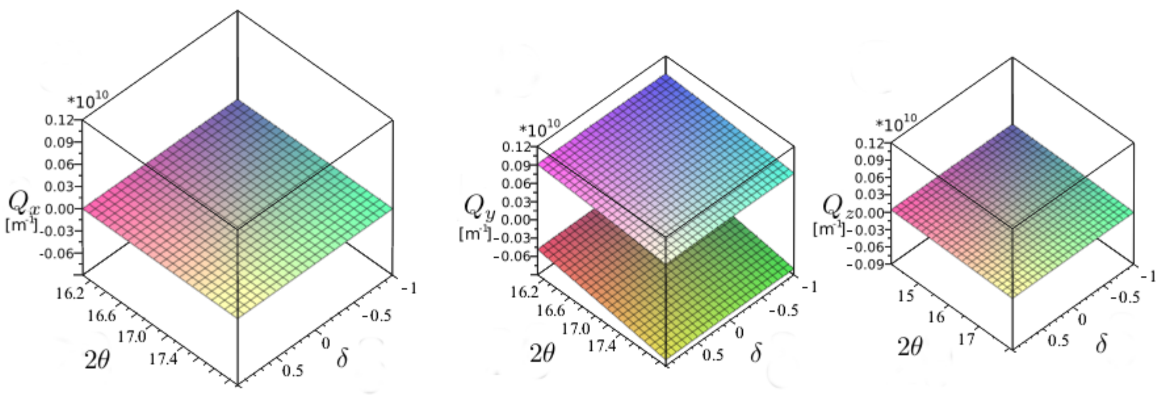

Supplementary Figure 1: Calculated Q_x,Q_y,Q_z values for the detector area (a) Difference in Q_x between the first and the last angle of the motion in θ. (b) Q_y values for the first and the last angle of the motion in θ. (c) difference in Q_z between the first and the last angle of the motion in θ. In panel (a) and (c) we show that the Q_z and Q_x values of the detector do not change during the rocking of the sample. In panel (b) we have two different planes of Q_y for the first and last scan. The detector integrates over the volume between the planes during the scan. This gives the range of Q_y over which we integrate during each scan.

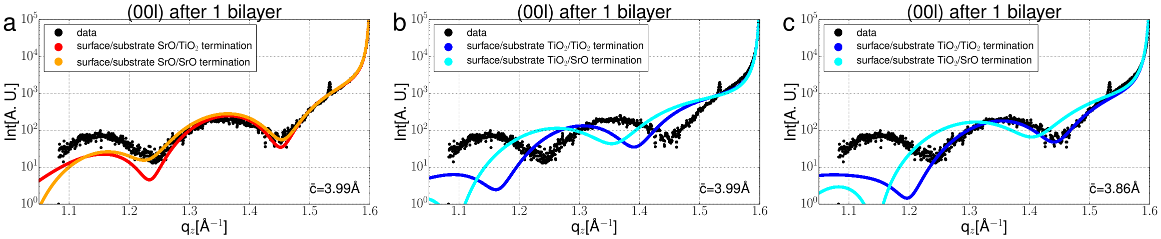

Supplementary Figure 2: Surface Termination from the first Bilayer The figure shows the intensity vs. Q_z data (black) for the 2/6 BTO/STO on STO superlattice after the growth of one bilayer. (a) shows fits for a SrO surface termination of the superlattice and TiO_2 termination (red), SrO termination (orange) of the substrate, the superlattice is fitted with an average out of plane lattice parameter of 3.99Å. One can see that these are good fits to the measured data, but they do not allow one to determine which of the two substrate surface terminations is present. (b) shows fits for a TiO_2 surface termination of the superlattice and TiO_2 termination(blue), SrO termination(cyan) of the substrate, the superlattice is fitted with an average out of plane lattice parameter of 3.99Å. These fits do not fit the data well. (c) shows fits for a TiO_2 surface termination of the superlattice and TiO_2 termination(blue), SrO termination(cyan) of the substrate, the superlattice is fitted with an average out of plane lattice parameter of 3.86Å. The blue fit is good but the average out of plane lattice parameter is smaller than the lattice parameters of STO and BTO which makes this fit unrealistic.

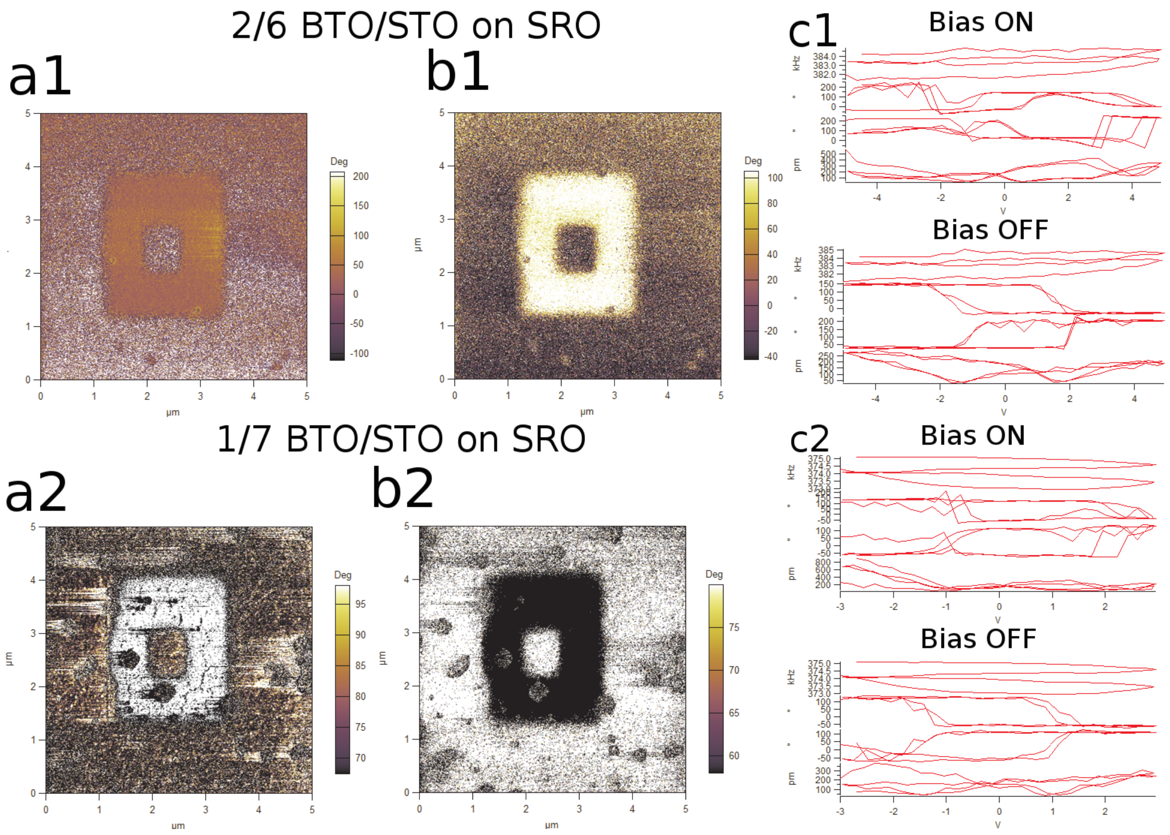

Supplementary Figure 3: phase and pfm data after writing domains together with single point PFM hysteresis loops for 2/6 and 1/7 BTO/STO superlattices The top panels (a1), (b1), (c1) show PFM data for the 2/6 BTO/STO grown on SRO sample. The bottom panels (a2), (b2), (c2) show PFM data for the 1/7 BTO/STO grown on SRO sample. (a1), (a2) Phase 1 data, (b1), (b2) Phase 2 date taken with DART PFM. The data was taken after writing the square with a constant bias into the superlattices. (c1), (c2) Single point PFM measurements in which the phase hysteresis and butterfly loops in the piezoelectric response can be seen. Both show that the measured samples are ferroelectric at room temperature.

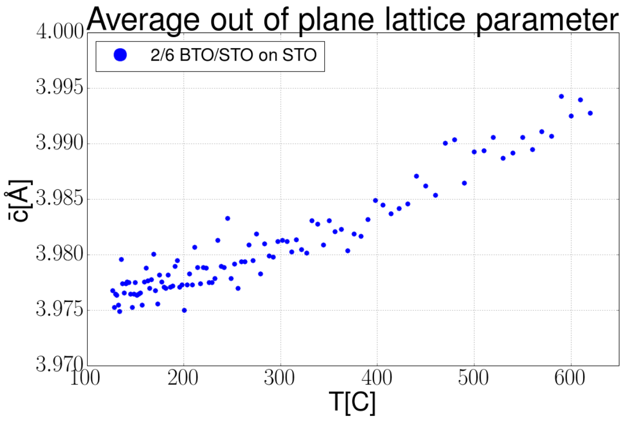

Supplementary Figure 4: $\bar{c}$ measured as a function of temperature for a 2/6 BTO/STO on STO superlattice

The figure shows the average out of plane lattice parameter $\bar{c}$ during the cooling of a 2/6 BTO/STO on STO superlattice. The lattice parameter gradually reduces while the sample is cooled indicating that no ferroelectric phase transition occurs. If a phase transition did occur there should be a sharp change in $\bar{c}$ at that temperature.

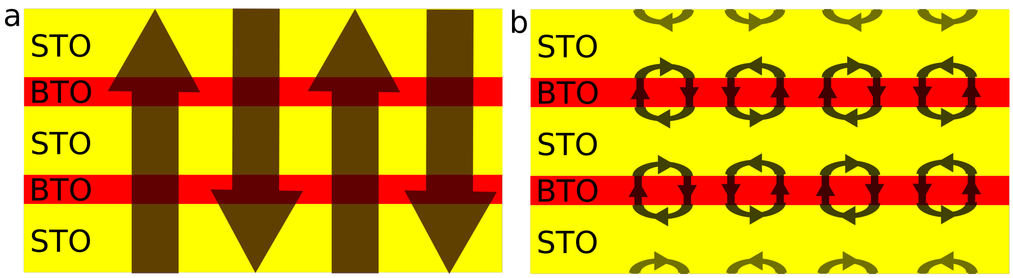

Supplementary Figure 5: The two domain coupling scenarios used to fit the data

(a) The homogeneous polarization model, in which the BTO and STO layers carry the same amount of polarization (b) the only BTO polarized model, in which only BTO layers are polarized out of plane and the polarization loops are closed inside the STO layers.

Supplementary Note 1: Growth rates

From the reflectivity data we can extract the growth rates per unit cell for STO grown on STO as (66.6 ± 2.0)s/unit cell, BTO grown on STO as (43.3 ± 1.3)s/unit cell for the first four layers and increasing after that. Within the BTO/STO superlattice the BTO growth rate is (44.5 ± 3.6)s/unit cell which is the same as for BTO on STO. The average growth rate for STO within the superlattice is not the same as when it is grown directly on STO. The measured average growth rate is (74.2 ± 7.6)s/unit cell, but a closer inspection reveals that the growth rate is different for odd unit cells, which take (80.2 ± 5.7)s/unit cell to complete and even unit cells which take (68.2 ± 3.2)s/unit cell. We believe that this is caused by the STO in the superlattice growing with a SrO termination (coming from growing on BaO terminated BTO surfaces), in contrast to the TiO_2 termination it normally has when growing directly on the substrate.

Supplementary Note 2: Scan calculations

To better illustrate the used scanning method we calculated the Q_x,Q_y and Q_z values for the detector area during the rocking of the θ motor. Supplementary Fig. 1 (a) shows the difference in Q_x between the first and the last angle of the scan. One can see that the Q_x value for each pixel of the detector does not change during the scan. The same is true for the Q_z values of each pixel (see Supplementary Fig. 1 (c)). The only direction in which one integrates during this kind of scan is the Q_y direction. This is shown in Supplementary Fig. 1 (b). During the scan each pixel integrates over the Q_y values between the first and last plane. This corresponds to a vertical line in the Q_y direction in Supplementary Fig. 1 (b).

Supplementary Note 3: Surface termination

The CTR of the (0 0 1) peak depends strongly on the surface termination of the heterostructure(see Supplementary Fig. 2). We demonstrate this with the examples of the first bilayer of a 2/6 BTO/STO superlattice grown on STO. This heterostructure contains 6 unit cells STO on top of 2 unit cells BTO and is grown on a STO substrate. There are two possible surface terminations for this example. One is the TiO_2 surface termination, which is the expected surface termination of the substrate after chemical treatment. The

other one is SrO surface termination. Our fit for the SrO surface termination for the superlattice and TiO_2 termination(red), SrO termination(orange) of the substrate is shown in Supplementary Fig. 2 (a). One can see that these are good fits to the measured data but one can not distinguish between the two substrate surface terminations. The superlattice is fitted with an average out of plane lattice parameter of 3.99Å. When we simulated the same superlattice (with the same average out of plane lattice parameter of 3.99Å) with a TiO_2 surface termination of the superlattice and TiO_2 termination(blue), SrO termination(cyan) of the substrate Supplementary Fig. 2 (b), we could not observe a good match with the taken data. Finally we tried to fit the superlattice with a TiO_2 surface termination of the superlattice and TiO_2 termination(blue), SrO termination(dark blue) of the substrate Supplementary Fig. 2 (c). These fits lead to an average out of plane lattice parameter of 3.86Å, which is smaller than the lattice parameters of STO and BTO and makes this fit unrealistic. This leads to the conclusion that the superlattice has a SrO surface termination after the first bilayer. By changing the growth parameters one might be able to change the surface termination.

Supplementary Note 4: Piezo Force Microscopy

To show that the samples are ferroelectric at room temperature Piezo Force Microscopy (PFM) was performed on the samples with a SRO back electrode. First a square pattern domain was written onto the sample by applying a constant bias of 5V to the sample in the square area. Afterwards the domains were read out using Dual AC Resonance Tracking(DART) PFM. The experiment was performed on an Asylum MFP3D Atomic Force Microscope(AFM). Supplementary Fig. 3 shows the taken data panels (a1), (b1), (c1) are for the 2/6 BTO/STO sample and panels (a2), (b2), (c2) are for the 1/7 BTO/STO sample. The phase 1 signal is shown in Supplementary Fig. 3 (a1), (a2). The areas of the sample which are polarized in different directions show a clear phase shift. One can observe the phase shift for the phase 2 signal in Fig. Supplementary 3 (b1), (b2), where the high and low phase signal between the domains switched. This shows the correct tuning of the instrument. Supplementary Fig. 3 (c1), (c2) show point PFM measurements, one can see the phase hysteresis and butterfly loops in the piezoelectric response. To connect the room temperature PFM with the high temperature x-ray data which is the main focus of the

paper we show that on cooling the lattice parameter decreases smoothly with no evidence of a ferroelectric phase transition between room temperature and 650°C. (See Supplementary Fig. 4

Supplementary Note 5: Domain coupling models

To help illustrate the two domain coupling models used to fit the experimental data we have included rough schematics. (See Supplementary Fig. 5) The first case is extremely strong coupling between layers (Supplementary Fig. 5 (a)), leading to a homogeneous polarization model. In this model polarization can be considered homogeneous throughout the superlattice, with both materials (BTO and STO) having the same polarization value. This results in a polar dependence for both $c_{BTO}(P)$ and $c_{STO}(P)$, according to conventional strain polarisation coupling relationships. In the other extreme, in which layers are essentially decoupled (Supplementary Fig. 5 (b)), the ferroelectric material, BTO, is polarized and the dielectric material, STO, has no polarization, ie. an only BTO polarized model. This time only c_{BTO}(P) depends on the polarization and c_{STO} is independent of the polarization.
